# Stomatal closure is induced by hydraulic signals and maintained by ABA in drought-stressed grapevine

**DOI:** 10.1038/srep12449

**Published:** 2015-07-24

**Authors:** Sergio Tombesi, Andrea Nardini, Tommaso Frioni, Marta Soccolini, Claudia Zadra, Daniela Farinelli, Stefano Poni, Alberto Palliotti

**Affiliations:** 1 Dipartimento di Scienze Agrarie, Alimentari e Ambientali, University of Perugia, Borgo 20 giugno 74, 06121 Perugia, Italy; 2 Dipartimento di Scienze della Vita, University of Trieste, Via L. Giorgieri 10, 34127 Trieste, Italy; 3 Dipartimento di Scienze Farmaceutiche, University of Perugia, Borgo 20 giugno 74, 06121 Perugia, Italy; 4 Dipartimento di Scienze delle Produzioni Vegetali Sostenibili, Università Cattolica del Sacro Cuore, Via E. Parmense 84, 29100 Piacenza, Italy

## Abstract

Water saving under drought stress is assured by stomatal closure driven by active (ABA-mediated) and/or passive (hydraulic-mediated) mechanisms. There is currently no comprehensive model nor any general consensus about the actual contribution and relative importance of each of the above factors in modulating stomatal closure *in planta*. In the present study, we assessed the contribution of passive (hydraulic) vs active (ABA mediated) mechanisms of stomatal closure in *V. vinifera* plants facing drought stress. Leaf gas exchange decreased progressively to zero during drought, and embolism-induced loss of hydraulic conductance in petioles peaked to ~50% in correspondence with strong daily limitation of stomatal conductance. Foliar ABA significantly increased only after complete stomatal closure had already occurred. Rewatering plants after complete stomatal closure and after foliar ABA reached maximum values did not induced stomatal re-opening, despite embolism recovery and water potential rise. Our data suggest that in grapevine stomatal conductance is primarily regulated by passive hydraulic mechanisms. Foliar ABA apparently limits leaf gas exchange over long-term, also preventing recovery of stomatal aperture upon rewatering, suggesting the occurrence of a mechanism of long-term down-regulation of transpiration to favor embolism repair and preserve water under conditions of fluctuating water availability and repeated drought events.

Stomatal regulation is one of the key mechanisms allowing plants to regulate and optimize CO_2_ assimilation versus evaporative water loss. Under conditions of soil water limitation and/or high atmospheric evaporative demand, partial or complete stomatal closure allows plants to maintain a favorable water balance while limiting the carbon gain^1^,^2^,^3^,^4^. Stomatal opening responds to several environmental and physiological factors such as light, carbon dioxide concentration, leaf-to-air vapor pressure deficit, leaf/plant water status and abscisic acid (ABA)^5^. However, there is currently no comprehensive model nor any general consensus among scientists, about the actual contribution and relative importance of the above factors in modulating stomatal closure *in planta*.

When water availability becomes limiting for plant physiological processes, stomatal closure acts as an early response buffering the drop of xylem water potential and the consequent risk of massive xylem embolism and catastrophic hydraulic failure^6^,^7^. Salleo *et al.*^8^ have reported that the onset of partial stomata closure in transpiring *Laurus nobilis* L. plants shows temporal coincidence with stem water potential values approaching the cavitation threshold, as revealed by the concurrent recording of ultrasound acoustic emissions produced by stems and leaves^9^. Taking into account that stomatal conductance to water vapour is generally co-ordinated with liquid phase hydraulic conductance of the soil-to-leaf pathway^10^,^11^,^12^, these findings have suggested that stomatal aperture is primarily modulated by hydraulic signals^13^,^14^. On the other hand, several studies have stressed the prominent role played by ABA production/accumulation in modulating stomatal closure^15^,^16^,^17^,^18^. ABA is thought to be synthesized under water stress conditions either at the root or leaf level, and it would lead to the depolarization of guard cell membranes triggering osmotic ion efflux and the loss of guard cell turgor^19^,^20^,^21^,^22^,^23^.

Recently, Brodribb and McAdam^24^ suggested that stomatal closure under leaf water deficit has evolved from a passive hydraulic process mediated by water potential changes to an active process controlled by the extrusion of anions from guard cells. In support to this view, the ancient lineages of lycophytes and ferns lack of active control mechanism to modulate stomatal closure^24^, while in the gymnosperm *Metasquoia glyptostroboides* ABA effect is additive to the passive hydraulic influence on stomatal closure^25^.

In some angiosperm species, a lack of correlation between changes in foliar ABA and onset of stomatal closure has been reported^26^,^27^,^28^,^29^. *Vitis vinifera* is considered as a model species for studying responses to drought stress of woody crops^30^. In this species, large increases in foliar ABA have been reported under drought stress conditions. On this basis, ABA was suggested to be a major factor inducing stomatal closure in grapevine^31^,^32^,^33^,^34^. Different levels of foliar ABA accumulation were also suggested to underlie different stomatal behaviours in *V. vinifera* cultivars displaying near-isohydric or anisohydric hydraulic strategies^35^. However, recent studies have reported that near-isohydric cultivars and anisohydric cultivars also display different vulnerabilities to xylem cavitation, and it has been suggested that the different stomatal behaviour of grapevine genotypes under water limitation might indeed be primarily related to their different hydraulic vulnerabilities, tailoring to ABA production and accumulation patterns a secondary role^36^. Outcomes of these research efforts suggest that stomatal closure in grapevine might be induced by a combination and/or an additive effects of hydraulic (water potential-mediated) and chemical (ABA-mediated) mechanisms. While *in vitro* ABA supply to leaves causes a rapid stomata closure^19^,^33^,^37^,^38^, it is not clear whether ABA accumulation in the leaf *in planta* is actually the trigger of stomata closure or just an additive signal involved in the long-term maintenance of stomatal closure under prolonged drought and/or under the initial phases of post-drought recovery. Indeed, studies on other species reported that stomatal behavior after drought is likely influenced by a combination of hydraulic and non-hydraulic factors^25^,^39^.

The aim of the present study was to determine the incidence of passive (hydraulic) and active (ABA mediated) mechanisms of stomatal closure in *V. vinifera* plants facing drought stress, and to determine which is the signal triggering stomatal closure when vines are exposed to limiting water conditions. Our hypothesis was that in *V. vinifera* stomatal closure is primarily triggered by water potential decrease and coordinated with xylem vulnerability to embolism formation, with leaf ABA content eventually increasing after severe reduction of stomatal conductance to prevent sudden stomatal reopening upon transient plant rehydration. In this study we used Sangiovese and Montepulciano, two of the most important grapevine cultivars, that are considered anisohydric and isohydric, respectively.

## Results

**Figure 1:**
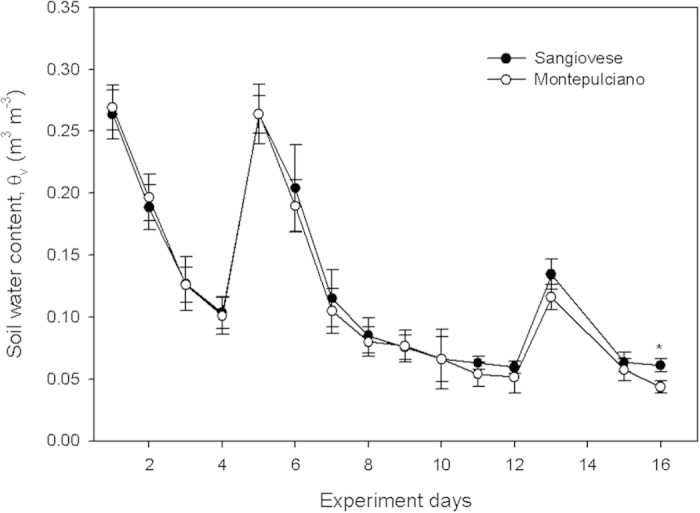
Soil water content in the pots of Sangiovese and Montepulciano vines during the experiment. Each point is the mean of three pots ± SE. Points with asterisk are different per P < 0.05 (t-student test).

**Figure 2:**
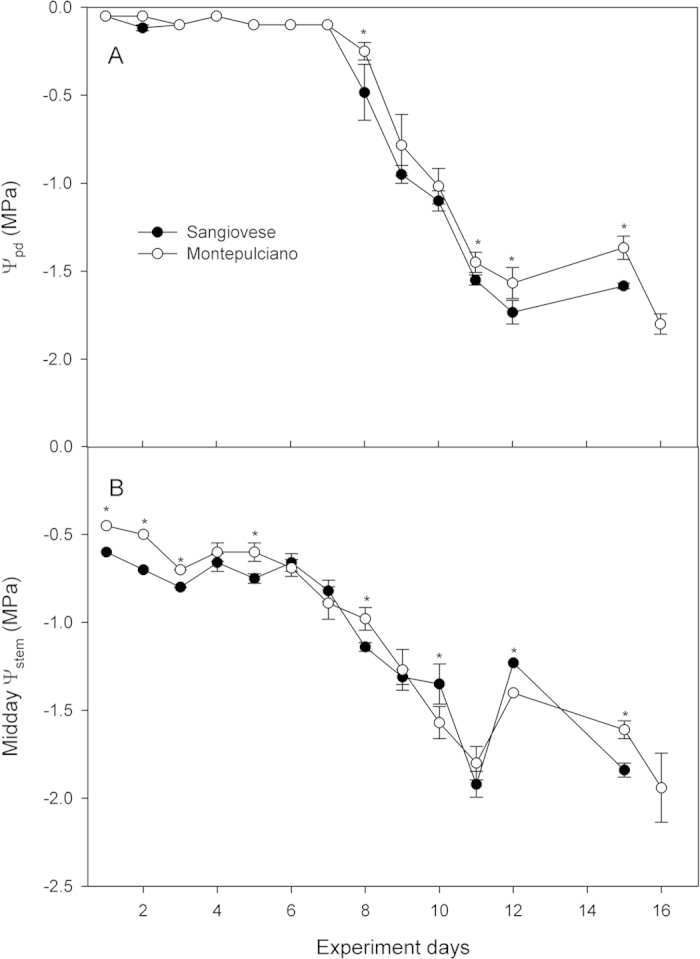
Ψ_pd_ (**A**) and Ψ_stem_ at midday (**B**) of Sangiovese and Montepulciano grapevine during the experiment. Each point is the mean of five vines ± SE. Points with asterisk are different per P < 0.05 (t-student test).

Soil water content (Θ_w_) decreased progressively during the period when drought was imposed (Fig. 1). Two heavy rain (>50 mm) events occurred during the experiment (on the afternoon of day 4 and in the late morning of day 12), and partial soil water content recovery occurred in particular on day 4. The minimum values of Θ_w_ recorded were between 0.05 and 0.07 m^3^ m^−3^. No significant differences were observed between the two cultivars in terms of soil water content except on day 16. Pre-dawn water potential was similar in the two cultivars during the first 7 days of the experiment (Fig. 2A). From day 8 until the end of the experiment, Sangiovese had slightly lower Ψ_pd_ than Montepulciano, and in days 8, 11, 12 and 15 the difference recorded was statistically significant (P < 0.05). Midday stem water potential (Ψ_md_) decreased progressively during the experiment and was lower in Sangiovese than in Montepulciano excluding days 4, 6, 7, 9, and 11 when the difference was not significant, or days 10 and 12 when Sangiovese had significantly higher Ψ_md_ than Montepulciano (Fig. 2B).

**Figure 3:**
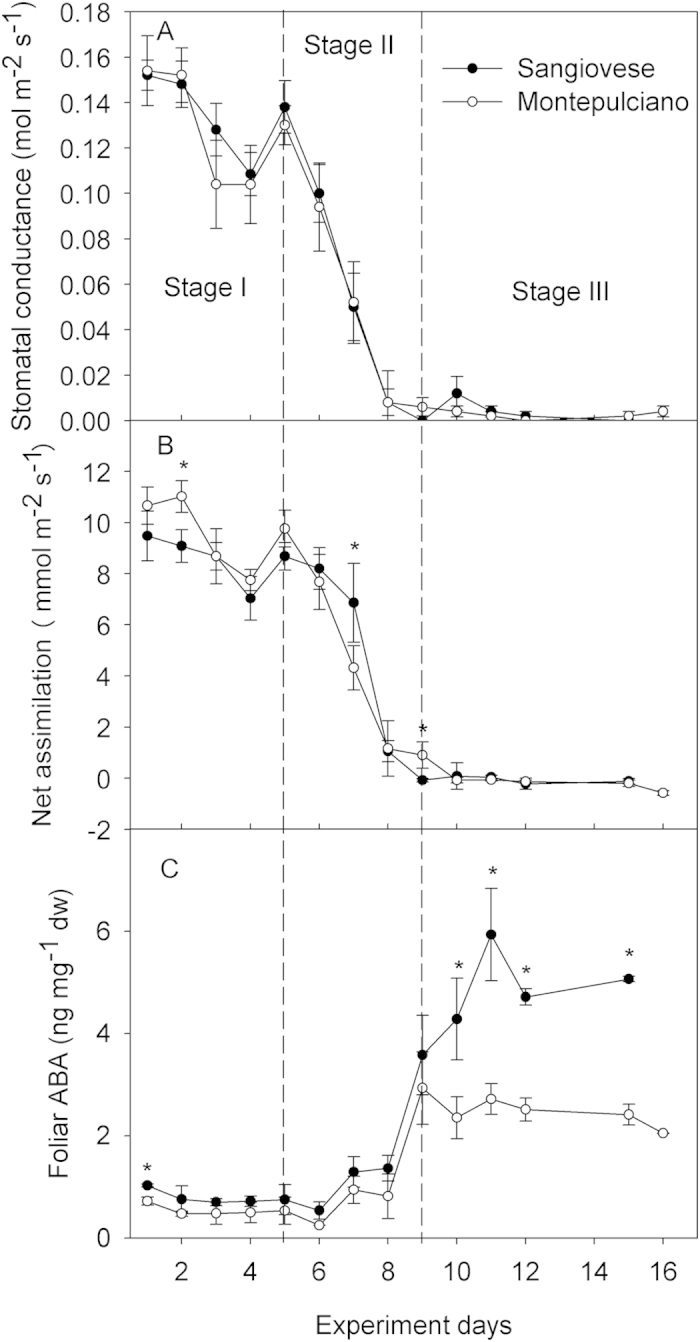
Stomatal conductance (**A**), net CO_2_ assimilation (A_n_) (**B**) and leaf [ABA] (**C**) measured at midday during the experiment. Each point is the mean of five vines ± SE. Points with asterisk are different per P < 0.05 (t-student test).

Stomatal conductance (g_s_) measured at midday decreased during the experiment showing a similar trend between cultivars (Fig. 3A). In more detail, the largest g_s_ decrease occurred between days 5 and 8 when stomatal conductance dropped from about 0.130 mol m^2^ s^−1^ to 0.008 mol m^2^ s^−1^ in both cultivars. Net assimilation (A_n_) rates followed a pattern similar to g_s_ (Fig. 3B), yet over the first days of the experiment Montepulciano had higher assimilation rates than Sangiovese (days 1 and 2), whereas in the second part of the experiment, when g_s_ was limited and stem water potential decreased (after day 7), Montepulciano and Sangiovese had similar net assimilation values. Foliar ABA remained constant (mean values per cultivar were not significant, P > 0.05, ANOVA) from day 1 until day 8 (Fig. 3C). On day 9, Foliar ABA increased about 3 fold in both cultivars and in Sangiovese it continued to rise until day 11, when foliar ABA peaked to a value almost 6 fold higher as compared to initial values.

**Figure 4:**
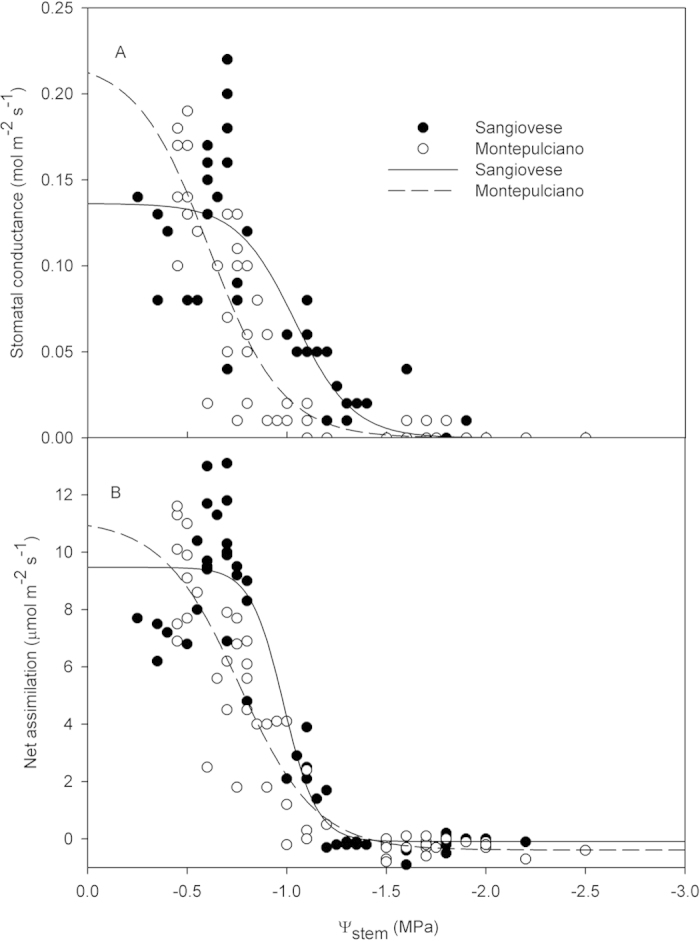
Relationship between midday stomatal conductance (**A**), midday net CO_2_ assimilation (A_n_) (**B**) and midday Ψ_stem_ in Sangiovese and Montepulciano vines.

Stomatal conductance and net assimilation rates showed a faster decline at higher Ψ_md_ (i.e. less negative) in Montepulciano than in Sangiovese (Fig. 4A). In both cultivars, g_s_ (Sangiovese R^2^  =  0.75 P  <  0.001; Montepulciano R^2^ = 0.83 P < 0.001 ANOVA) and A_n_ (Sangiovese R^2^ = 0.90 P < 0.001; Montepulciano R^2^ = 0.87 P < 0.001 ANOVA) were significantly correlated to Ψ_md_. In particular, g_s_ started to decline in Montepulciano at Ψ_md_ < −0.7 MPa and reached values close to zero at Ψ_md_ < −1.1 MPa, while, in Sangiovese, g_s_ started to decline at Ψ_md_ < −1.0 MPa and reached values close to zero at Ψ_md_ < −1.5 MPa. Similar trends were observed in leaf A_n_ in both cultivars, with Montepulciano displaying a steeper reduction of A_n_ at progressively lower Ψ_md_ than Sangiovese (Fig. 4B).

**Figure 5:**
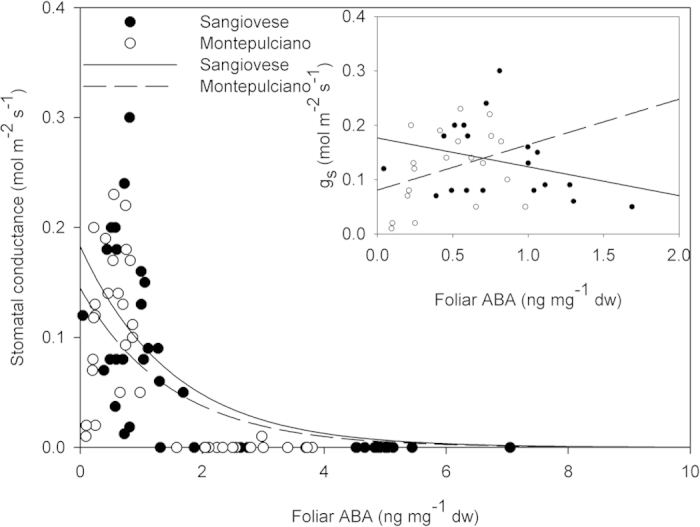
Stomatal conductance *vs* leaf [ABA] in Sangiovese (y = 0.18 × e^−0.66x^, R^2^ = 0.48, P < 0.001) and Montepulciano (y = 0.14 × e^−0.67x^, R2 = 0.42, P < 0.001) vines measured at midday during the experiment. The insert depict stomatal conductance (g_s_) *vs* foliar ABA per g_s_ > 0 in Sangiovese (R^2^ = 0.09, P = 0.20) and Montepulciano (R^2^ = 0.11, P = 0.15).

Although g_s_ was correlated with foliar ABA by an exponential correlation (Sangiovese, y = 0.18 × e^−0.66x^, R^2^ = 0.48 P < 0.001; Montepulciano y = 0.14 × e^−0.67x^, R^2^ = 0.42 P < 0.001), there was no significant correlation between g_s_ and foliarABA at g_s_ > 0 (Sangiovese R^2^ = 0.09, P = 0.20; Montepulciano R^2^ = 0.11, P = 0.15) (Fig. 5).

**Figure 6:**
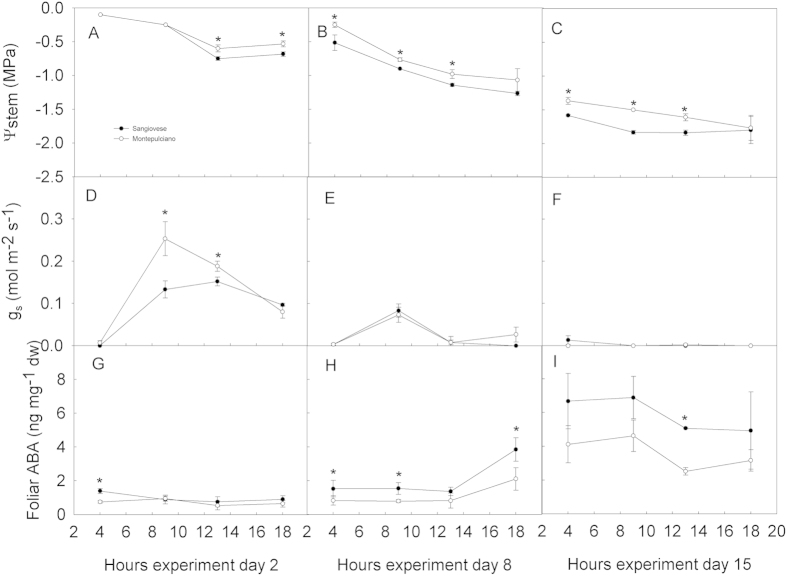
Daily course of Ψ_stem_, stomatal conductance, foliar ABA in day 2 (A,D,G), 8 (B,E,H) and 15 (C,F,I) in Montepulciano and Sangiovese vines. Each point is the mean of five vines ± SE. Points with asterisk are different per P < 0.05 (t-student test).

Diurnal course of Ψ_stem_, g_s_ and foliarABA progressively changed depending on water stress severity (Fig. 6). On day 2, Ψ_stem_ decreased from 4:00 am to 1:00 pm and remained approximately stable until 6:00 pm. During the same day, maximum g_s_ was measured at 9:00 AM while a progressive decrease was observed during the rest of the day. Montepulciano displayed higher g_s_ values than Sangiovese at all daytimes except at 6:00 pm. Foliar ABA was constant over the whole day and was slightly higher in Sangiovese than in Montepulciano, although this difference was statistically significant only at 4:00 am. On day 8, Ψ_stem_ decreased from −0.3 MPa and −0.5 MPa at pre-dawn (4:00 am), to −1.0 MPa and −1.2 MPa at 6:00 pm in Montepulciano and Sangiovese, respectively. Ψ_stem_ measured at 6:00 pm was slightly more negative than values measured at 1:00 pm. Montepulciano had Ψ_stem_ significantly higher than Sangiovese over the whole day. On day 8, g_s_ was consistently lower than on day 2 and maximum g_s_ measured at 9:00 am was below 0.1 mol m^−2^ s^−1^, while near-complete stomatal closure occurred at 1:00 pm and was maintained also at 6:00 pm. On the same day, foliar ABA was slightly higher than values recorded on day 2, although there was no difference between the measurements carried out at 4:00 am, 9:00 am and 1:00 pm on day 2 and day 8. On day 8, a consistent increase of foliar ABA was measured at 6:00 pm in both cultivars. On day 15, Ψ_stem_ decreased from −1.36 MPa and −1.58 MPa at pre-dawn (4:00 am) to −1.76 MPa and −1.8 MPa at 6:00 pm in Montepulciano and Sangiovese, respectively. In Montepulciano, Ψ_stem_ was consistently higher than in Sangiovese at any timing except for 6:00 pm. On the same date, g_s_ was steadily close to zero in both cultivars. Foliar ABA further increased in both cultivars on day 15 compared to day 8. Although the difference was not statistically significant during the day except at midday, Sangiovese leaves had higher ABA than Montepulciano leaves on day 15.

**Figure 7:**
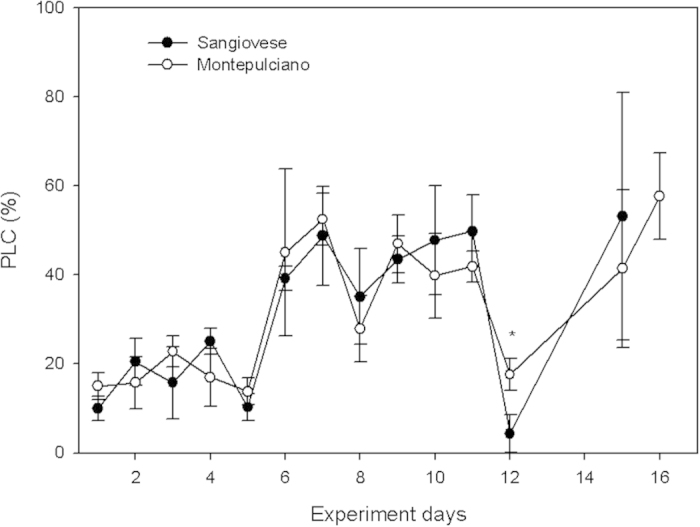
PLC of Sangiovese and Montepulciano petioles measured at midday during the experiment. Each point is the mean of five vines ± SE. Points with asterisk are different per P < 0.05 (t-student test).

Leaf petiole percent loss of hydraulic conductance (PLC) ranged across 20% until day 5 in both cultivars (Fig. 7). On day 6, in both cultivars, leaf petiole PLC increased to values close to 50% and remained stable until day 11. On day 12, when petiole PLC was measured immediately after rain, petiole PLC dropped to values below 20%. On this date, Montepulciano had significantly higher PLC than Sangiovese. On day 15 and 16, PLC increased again up to values similar to those measured between days 6 and 11.

**Figure 8:**
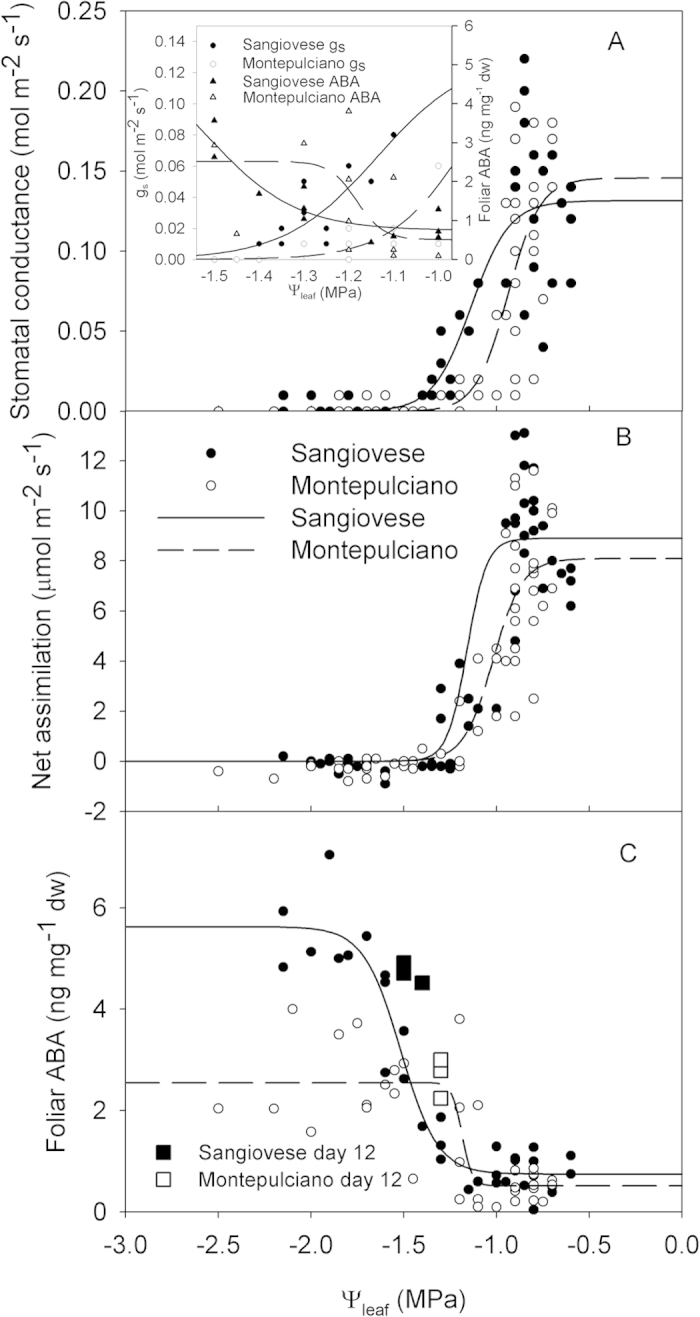
Relationship between midday stomatal conductance (**A**), midday net CO_2_ assimilation (A_n_) (**B**), foliar ABA (**C**) and Ψ_leaf_ in Sangiovese and Montepulciano vines. The insert depict stomatal conductance (g_s_) and foliar ABA when stomata closure occurred in the interval −1 MPa < Ψ_leaf_ < −1.5 MPa.

Foliar ABA, as well as g_s_ (Sangiovese R^2^ = 0.67 P < 0.001; Montepulciano R^2^ = 0.71 P < 0.001 ANOVA) and A_n_ (Sangiovese R^2^ = 0.79 P < 0.001; Montepulciano R^2^ = 0.80 P < 0.001 ANOVA), were significantly correlated with Ψ_leaf_ (Sangiovese R^2^ = 0.87 P < 0.001; Montepulciano R^2^ = 0.62 P < 0.001 ANOVA) (Fig. 8). Foliar ABA increased at water potential values at which g_s_ was below 0.02 mol m^−2^ s^−1^ in both cultivars.

## Discussion

*Vitis vinifera* is generally considered an anisohydric species, although intra-specific variability of stomatal behaviour under water limitation condition has been observed in different grapevine genotypes^40^. As also reported for other species^41^, progressive dehydration imposed on Sangiovese and Montepulciano vines induced physiological responses that can be conveniently divided in three successive stages (Fig. 3). At stage I, when soil water is still plenty available and not affected by the onset of drought, gas exchange rates are not influenced by water shortage. In stage II, the progressive reduction of soil available water induces partial stomatal closure and gas exchange limitation. In stage III, stomata are fully and steadily closed, gas exchange rates drop to values close to zero and leaf senescence is triggered.

Throughout stage I the vine can still extract water from soil, so that no stomatal regulation of transpiration is apparent and diurnal changes of Ψ_stem_ are mainly influenced by the plant hydraulic resistance and the atmospheric evaporative demand. In our experiment, at this stage, PLC due to embolism was negligible (<20%) and leaf net assimilation was maximized. When soil water content approached critical values (<0.1 m^3^ m^−3^) in stage II, plant water potential decreased, probably enhancing the plant capacity to extract water from the soil. Progressive drop of plant water potential, however, causes xylem pressure to drop to very negative values, thus increasing the likelihood of embolism induction in xylem vessels and consequent hydraulic failure^7^. Under such conditions, stomatal closure plays a key role in preventing hydraulic failure by regulating the rate of water loss and limiting the xylem pressure drop^6^,^8^,^16^. In our experiment, stomata started to close 6 days after the beginning of water withdrawal, concurrently with the recorded increase of PLC of leaf petioles^42^ that approached values close to 50%. In particular, gas exchange rates were more limited in the afternoon than in the morning, when plant water status was more favourable and PLC was likely lower due to partial nocturnal vessel refilling^42^,^43^,^44^. These results are consistent with the coordination between stomatal movements and PLC reported in other species: in walnut Cochard *et al.*^45^ concluded that cavitation avoidance was a physiological function associated with stomatal regulation during water stress; in douglas-fir, g_s_ was linearly correlated with PLC^46^; in a study on eight tropical species Brodribb *et al.*^47^ concluded that leaf and xylem hydraulic traits are correlated with the response of stomata to Ψ_leaf_ inducing loss of xylem hydraulic conductivity; and finally, in a literature overview on 70 woody species, Klein^48^ concluded that stomatal sensitivity to leaf water potential strongly relates to xylem characteristics.

ABA has been frequently suggested to act as a root-to-leaf chemical signal under progressive soil drying^49^, but some experimental observations apparently contrasts with this view. As an example, Soar *et al.*^50^ recorded gradients in xylem and foliar ABA along shoots of *V. vinifera*, and found that the concentrations of the hormone were higher close to the apex and decreased downwards. In our experiment, foliar ABA remained below 2 ng mg^−1^ dw up to the occurrence of almost complete stomata closure (8^th^ day). A significant increase of foliar ABA occurred only after complete stomatal closure had already occurred. On day 7 foliar ABA rose by 63% (Sangiovese) and 74% (Montepulciano) in comparison with the mean foliar ABA recorded in the previous days. However, we note that these data need to be interpreted with caution, as our experimental approach cannot rule out the hypothesis that early stomatal responses are influenced by very localized increases in ABA concentration, eventually below the detection limit of bulk measurements. Moreover, our data do not exclude the possibility of very local changes in ABA uptake rate into guard cells.

In grapevine leaves fed with exogenous ABA, a similar foliar ABA increase resulted in g_s_ reduction of about 20%^33^, i.e. less than the 64% and 61% reduction of g_s_ measured in our experiment in Sangiovese and Montepulciano, respectively. Furthermore, foliar ABA was correlated with Ψ_leaf_ and increased at Ψ_leaf_ values corresponding to g_s_ < 0.02 mol m^−2^ s^−1^, thus suggesting a potential signal inducing ABA synthesis and mediated by water potential. These data support the hypothesis of foliar ABA accumulation when stomata are nearly or completely closed. These results are consistent with those reported in some gymnosperms and other angiosperm species. As an example, in *M. glyptostroboides,* ABA production was triggered after leaf turgor loss and stomatal closure^25^. In progressively water stressed *Zea mays* and *Sorghum bicolor,* stomatal closure preceded any detectable ABA increase^26^, while in flooded tomato plants, bulk leaf ABA increased only after the onset of stomatal closure^29^. Finally, in stress adapted cotton plants (*Gossypium hirsutum* L.) higher levels of ABA were not correlated with stomatal response to low leaf water potentials^27^ and in one *Populus* spp. hybrid, stomatal aperture was not affected by ABA^28^. In our study, when stomatal conductance was plotted against foliar ABA, a significant non-linear correlation appeared (R^2^ = 0.47, P < 0.0001), but when non limiting (>0.05 mmol m^2^ s^−1^)^51^ and limiting stomatal conductance values were separately linearly regressed, no significant correlation was observed (Fig. 5). These results obtained during a relatively fast (2 weeks) dry-down experiment apparently contrast with those obtained by Speirs *et al.*^52^ on vines planted in the field subject to different irrigation treatments over the season.

Our results suggest reconsidering the role played by ABA in grapevines subjected to drought stress. Apparently, ABA played no critical role in regulating gas exchange during early onset of drought conditions, as stomatal closure was apparently triggered by xylem embolism and consequent reduction of plant hydraulic conductance and leaf turgor. After stomatal closure, ABA increased by 566% and 418% in Sangiovese and Montepulciano, respectively. These levels were well above those measured by Loveys^33^, who measured in ABA-fed grapevine leaves a 50% reduction of stomatal conductance reduction upon ABA increase by 366%. In our experiment, a five-fold rise appeared to be effective at constraining stomatal aperture even after recovery of plant water status. In fact, when a heavy rain event occurred on day 12, water availability in the soil increased to levels not limiting for stomatal conductance, and recovery of Ψ_stem_ and refilling of embolized vessels were also recorded. Nonetheless, stomatal conductance remained close to zero, and this suggests that ABA inhibited stomatal re-opening although water availability was sufficient to fuel the recovery of water potential and xylem conductance. Our results would also suggest that stomatal closure can trigger foliar ABA increase, and such high levels would persist afterwards despite transient recovery of leaf water status. Stomatal behaviour patterns similar to those described here were reported in *Pinus radiata* and *Eucalyptus pauciflora* by Brodribb and McAdam^24^ and Martorell *et al.*^39^, respectively. On the basis of these studies and our present results, it might be speculated that this behaviour might represent an effective physiological mechanism to prevent water loss in environments where plants are exposed to the risk of periodical summer drought, in that initial drought stress could induce, via ABA accumulation, the *a priori* down-regulation of leaf transpiration. Furthermore, stomatal closure induced by ABA has been indicated to favour vessel refilling^53^ by promoting de-polymerization of starch pools stored in parenchyma cells to produce sucrose or other sugars and generate the necessary osmotic gradients driving refilling^54^,^55^. The lack of stomatal conductance recovery after re-watering at day 12, coupled with the prompt decrease of PLC, would also suggest that stomatal closure induced by ABA is an important mechanism to favour fast rehydration and the rise of xylem pressure, thus further promoting osmotic-mediated embolism recovery in the case of occasional rewatering^56^,^57^.

Tallman^58^ (2004) hypothesized that diurnal patterns of stomatal movement are linked to ABA fluctuations and that stomatal movements are the results of the combination of ABA catabolism early in the morning and ABA biosynthesis and import from the apoplast around guard cells after midday. In our experiment, foliar ABA was steady during the whole day when water was not limiting. When stomatal closure was observed in the late morning of day 8, foliar ABA rose only in late afternoon. When stomata were steadily closed over the day (day 15) foliar ABA was steadily higher (in comparison to day 2) over the whole day. It has to be noted that foliar ABA levels might not be representative of levels actually active in guard cells. Locally active ABA levels result from the activity of ABA transport and the intensity of ABA metabolism. Therefore, levels of ABA in guard cells, which regulate turgor and are crucial for stomatal movements, could be increased over bulk leaf levels by spatially restricted activity of ABA transporters or ABA cleaving/conjugating/deconjugating enzymes. Our experimental procedures do not allow to detect eventual ABA accumulation patterns in guard cell that could explain stomatal movements during the day, but the analytical method used in the experiment allowed to measure the active form (not conjugated) of ABA. In agreement with previous experiments carried out on almond^59^, our data show that there is no foliar ABA reduction during the day time when stomata are open. These results are partially in contrast with the model proposed by Tallman^58^.

According to Tardieu and Simonneau^60^ and Soar *et al.*^35^, isohydric *vs* anisohydric behaviour (different stomatal closure dynamics in response to water potential drop) might arise from species-specific differences in stomatal sensitivity to ABA^49^,^61^. Sangiovese and Montepulciano are considered anisohydric and near-isohydric cultivars, respectively^36^,^62^,^63^. In our experiment, near-isohydric Montepulciano displayed lower levels of foliar ABA during all stages of the drought stress treatment in comparison with the anisohydric Sangiovese. Furthermore, the increase of Montepulciano foliar ABA recorded in stage III was less marked than in Sangionvese. Recent experiments pointed out that xylem vulnerability to cavitation might play a key role in defining stomata behaviour in *V. vinifera*^36^ and in a wide range of other species^48^. In particular, Klein^48^ concluded that isohydric *vs* anisohydric behavior do not represent two distinct categories but rather two extremes in a continuum of hydraulic strategies that dictates stomatal behavior due to the strong relationship between xylem characteristics and stomatal sensitivity to leaf water potential. Our results support this hypothesis, even though they do not allow to rule out a different sensitivity of stomata to similar levels of foliar ABA in these two cultivars.

Early studies on *Avena sativa* leaves pointed out that leaf senescence was triggered by stomatal closure^64^, and that stomatal closure itself triggers ABA accumulation, so that ABA might be the proximal cause of leaf senescence^65^. Experiments on rice, indicated that ABA is involved in senescence regulation^66^. Indeed, exogenously applied ABA induces the expression of several SAGs (senescence-associated genes) in *Arabidopsis thaliana*^67^. In a recent study on *A. thaliana*, Zhang and Gan^68^ concluded that there is a unique regulatory chain controlling stomatal movement and water loss during leaf senescence. Our results are consistent with this view, also considering that Sangiovese (which showed higher foliar ABA in comparison with Montepulciano) started leaf senescence and consequent leaf abscission earlier than Montepulciano (in the last experiment day it was not possible to perform measurements on Sangiovese because of the complete abscission of primary leaves).

In conclusion, in *V. vinifera* passive hydraulic control of stomatal closure appears to be dominant over eventual chemical signalling at the early phases of drought stress. The increase of foliar ABA concentration after stomatal closure was apparently addressed at inhibiting stomatal opening under transient rehydration, thus favouring embolism recovery. This mechanism could be effective in reducing *a priori* water loss in environments where periodical severe drought frequently occurs during the vegetative season. However, grapevine might also have evolved other, more sophisticated mechanisms to tightly control local ABA activity. More studies are needed to verify this alternative hypothesis.

## Materials and Methods

### Plant material

The study was conducted during July 2013 on 8-year-old potted *V. vinifera* vines of two top-grown red Italian cultivars i.e. Sangiovese (clone VCR30) and Montepulciano (clone R7), both grafted onto 1103 Paulsen rootstock and grown in an outdoor area close to the Dept. of Agricultural, Food and Environmental Sciences of the University of Perugia (Region of Umbria, central Italy, 42°58’N, 12°24’E, elevation 405 m a.s.l.). Pots (60 liters volume) were filled with loam soil. At the end of February, each vine was pruned to retain four spurs with two buds each. All shoots were oriented upright using suitable stakes. A total number of twenty vines was used in the experiment. Ten vines per cultivar were used and initially maintained at field capacity until 7^th^ July. Water was supplied every day at 8:00 pm. On 8^th^ July, drought was imposed on all vines by completely suspending irrigation and covering pot surface by plastic film. The drought treatment was continued until complete leaf abscission in both cultivars.

Daily measurements of soil water content were carried out at 4:00 am by a Diviner 2000 capacitance probe (Sentek Environ Tech., Sentek Environment Technologies, Stepney, South Australia), using access tubes located in three pots per cultivar. Measurements were performed at 100 mm, 200 mm and 300 mm depths from the soil surface of the pot (400 mm high). The total soil water content (Θ_w_) in the pot was expressed as the arithmetic mean of the measurements performed at different depths.

### Gas exchange and water potential

Stomatal conductance (g_s_) and net assimilation (A_n_) measurements were carried out on adult primary leaves grown between the 4^th^ and the 10^th^ node from the shoot base. Measurements were carried out between 4:00 am and 5:00 am, 8:00 am and 9:00 am, 12:00 am and 1:00 pm, 4:00 pm and 5:00 pm from 8^th^ July until 23^th^ July on one representative leaf sampled from 5 vines per cultivar using an open gas exchange system (ADC-System, LCA-3, Hoddesdon, UK) equipped with a Parkinson leaf chamber (11.2 cm^2^). Measurements during the daytime (8:00 am to 5 pm) were performed under saturating light conditions (PPFD > 1200 μmol photons m^−2^ s^−1^). Stem water potential (Ψ_stem_) was measured over the same days and daytimes using a pressure chamber (Soilmoisture Corp, Santa Barbara, CA, USA). Ψ_stem_ was measured on each vine on one mature leaf that had been wrapped in plastic film and aluminum foil 2 h prior to the measurements^69^. Water potential values measured at 4:00 am are reported as pre-dawn water potential (Ψ_pd_).

### Foliar ABA determination

Foliar ABA was determined on three primary leaves (the same used for gas exchange measurements) per cultivar sampled from different vines between 12 a.m. and 1 p.m. from 8^th^ July (day 1) to 23^rd^ July (day 16). On experimental days 2, 8 and 15 (9^th^, 15^th^ and 22^nd^ July, respectively), [ABA] was determined on leaves sampled concurrently with daytimes when gas exchange measurements were performed. ABA was extracted following the procedure described by Villarò *et al.*^70^ with some modifications. Leaves used for gas exchange measurements were immediately placed in liquid nitrogen and then stored in a freezer at −80 °C. Then, the material was weighted (fresh weight) and lyophilised (LIO5P, 5Pascal, Trezzano, Italy). Lyophilised material was weighted (dry weight) and grinded (MF10, IKAlabortechnik, Staufen, Germany). Leaf material (0.1 g) was extracted with 10 ml of methanol/water (1:1 v/v, pH = 3 with formic acid) for 30 min using a ultrasonic bath. After centrifugation, the supernatant was filtered through a paper filter and the same procedure was repeated for the remaining pellet. The collected filtrates were extracted twice with dichloromethane (15 ml) and the organic phase evaporated under vacuum. The residue was dissolved to a 1 ml with acetone and water/acetonitrile (50:50 v/v, 0.1% formic acid) for the HPLC analysis. Analytical standards of (±) Abscisic acid (purity ≥ 98.5%) was purchased from Sigma-Aldrich, PA-grade methanol, acetone, dichloromethane and formic acid, and HPLC-grade acetonitrile and water were purchased from VWR Chemicals. Analyses were performed on a Perkin-Elmer PE 200 system (Autosampler, Binary Pump and UV-VIS detector) equipped with an IB-Sil C8-HC (5 mm × 250 mm × 4.6 mm Phenomenex) column and IB-Sil C8 (5 mm × 30 mm × 4.6 mm Phenomenex precolumn) at a flow rate of 0.8 mL min^−1^; the injection volume was 20 μL and the detection was made at 270 nm. The mobile phase of acetonitrile/water (30:70 v/v, 0.1% formic acid) was previously filtered and degassed. The compound was identified by comparing the retention times with those of authentic reference compound. The peaks were quantified by an external standard method, using the measurements of the peak areas and a calibration curve. Stock solutions of ABA standards were prepared by diluting a solution (10 mg mL^−1^ in acetonitrile) to obtain a range of concentrations from 0.01 to 10 mg mL^−1^. The limit of detection (LOD) was 0.005 mg L^−1^.

### Percentage loss of hydraulic conductance

Percentage loss of xylem hydraulic conductance (PLC) was measured on five petioles per cultivar harvested between 12 a.m. and 1 p.m. on each day. Petioles were cut under water from leaves inserted nearby those used for gas exchange measurements. Hydraulic conductance of petioles was measured by connecting one sample end to plastic tubing filled with a filtered (0.2 μm) 20 mM KCl solution and connected to a pressure head maintained at a pressure (P) of 6 kPa. Flow (F) was measured by collecting the fluid from the distal end in pre-weighted sponge pieces fitted in plastic tubes. Flow readings were taken over 1 min time intervals. After approximately 30 minutes, once flow was found to be steady, sample hydraulic conductance (K) was calculated as F/P and the samples were flushed at P = 0.2 MPa for 30 min, to remove eventual embolism. After flushing samples, maximum hydraulic conductance (K_max_) was measured as above.

Percentage loss of hydraulic conductance (PLC) value was calculated as:


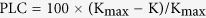


Differences between the two genotypes were assessed using the Student’s *t*-test (*P* < 0.05). The significance of regressions was tested using Pearson Product Moment Correlation.

## Additional Information

**How to cite this article**: Tombesi, S. *et al.* Stomatal closure is induced by hydraulic signals and maintained by ABA in drought-stressed grapevine. *Sci. Rep.* **5**, 12449; doi: 10.1038/srep12449 (2015).
